# Zoster Meningitis in an Immunocompetent Child after COVID-19 Vaccination, California, USA

**DOI:** 10.3201/eid2807.220600

**Published:** 2022-07

**Authors:** Sarah K. Daouk, Edwin Kamau, Kristina Adachi, Grace M. Aldrovandi

**Affiliations:** David Geffen School of Medicine at University of California, Los Angeles, USA

**Keywords:** zoster meningitis, COVID-19 vaccination, varicella zoster virus, papulovesicular rash, COVID-19, coronavirus disease, SARS-CoV-2, severe acute respiratory syndrome coronavirus 2, viruses, respiratory infections, zoonoses, vaccine-preventable diseases, California

## Abstract

Varicella zoster virus reactivation after COVID-19 vaccination has been reported in older or immunocompromised adults. We report zoster meningitis from live-attenuated varicella vaccine reactivation in an immunocompetent child after COVID-19 vaccination. This type of case is rare; COVID-19 and varicella vaccines remain safe and effective for appropriate recipients in the pediatric population.

The COVID-19 mRNA vaccines authorized in the United States are highly effective and safe, causing few adverse events (*1*). Cases of varicella zoster virus (VZV) reactivation after COVID-19 vaccination have been reported, but most occurred in older adults with comorbid conditions and known risk factors for VZV reactivation (*2*,*3*). We report a case of zoster meningitis reactivation from live-attenuated varicella vaccine (vOka) in a healthy child, occurring in close temporal relation with Pfizer-BioNTech (https://www.pfizer.com) BNT162b2 vaccination.

We evaluated a 12-year-old boy at the UCLA Mattel Children’s Hospital (Los Angeles, CA, USA) for papulovesicular rash with lumbar L1 dermatomal distribution; the rash gradually progressed to the L2 area (Figure), trunk, and scalp. The rash was preceded by a 1-week history of severe flank and thigh pain. The patient initially was seen at another hospital, where abdominal/pelvic computed tomography imaging led to a diagnosis of kidney stones and mesenteric adenitis. His pain persisted after discharge, and he then developed the vesicular rash described, along with twitching movements, headache, and photophobia. No change in mentation or focal neurologic findings were noted. The patient was otherwise healthy and taking no medications, including inhaled corticosteroids. His immunizations were up-to-date, including 2 doses of vOka, 1 at age 12 months and another at 18 months. He received his first dose of BNT162b2 vaccine 11 days before onset of the symptoms described.

**Figure:**
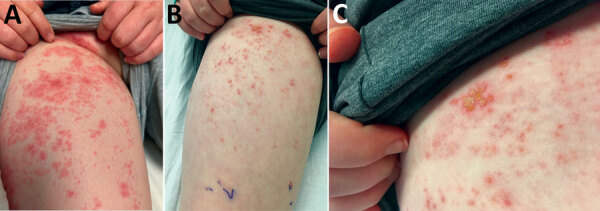
Rash in a 12-year-old boy with zoster meningitis after COVID-19 vaccination, California, USA. A) Rash on right groin and thigh at admission; B, C) improving rash after 4 days of acyclovir.

Laboratory studies revealed levels of leukocytes, platelets, C-reactive protein and transaminases within reference ranges. The cerebrospinal fluid had a leukocyte level of 252 cells/mm^3^ (reference range 0–5 cells/mm^3^) and a protein level of 96 mg/dL (reference range 15–45 mg/dL). Results of gram stain and cultures were negative. Cerebrospinal fluid and vesicular lesions were PCR-positive for VZV and were determined to be the vOka strain by the Centers for Disease Control and Prevention using a VZV fluorescence resonance energy transfer PCR. A cerebrospinal fluid viral meningoencephalitis antibody panel with >30 targets detected only VZV antibodies (Quest Diagnostics, http://www.questdiagnostics.com). The ePlex respiratory pathogen panel PCR (GenMark Diagnostics, https://www.genmarkdx.com) was positive for only rhinovirus/enterovirus, presumably representing recent infection. Other studies, including those for HIV, herpes simplex virus, SARS-CoV-2, and QuantiFERON-TB, were negative. Immunologic (T-cell, B cell, and natural killer cell cytotoxicity) studies revealed no underlying immunodeficiency. Exome sequencing performed using the Agilent SureSelect Clinical Research Exome XT kit (Agilent Technologies, https://www.agilent.com) and an Illumina HiSeq 2500 (Illumina, https://www.illumina.com) established no clinically important variants. The patient was treated with intravenous acyclovir for 10 days and recovered uneventfully.

The incidence of uncomplicated herpes zoster (HZ) in vaccinated children is rare: an estimated 48 cases/100,000 person-years compared with 230 cases/100,000 person-years in unvaccinated children, a 79% reduction (*4*). Rarer yet are cases where vOka reactivation led to meningitis; only 14 cases have been reported in children, 3 of whom were immunocompromised and 6 of whom had received the recommended 2 doses of vOKa (*5*). The average age of these patients was 12.5 years, and the time to HZ reactivation from their first vaccine dose averaged 11.5 years. Similarly, this patient experienced HZ reactivation when he was 12 years old, 11 years after when he received his first vOka dose, which was administered in his thigh. The L1/L2 location of his rash is consistent with vaccine-associated HZ, wherein the virus travels from the site of inoculation to establish latency in the lumbosacral plexus. In contrast, rash with HZ reactivation after infection occurs in the most common sites of vesicles, including the face and lower cervical or upper thoracic dermatomes (*6*). 

HZ reactivation is more frequent in adults, particularly in the elderly, because of waning of cellular immunity, and among immunocompromised persons. Changes in the immune status after COVID-19 vaccination has been postulated to lead to VZV reactivation (*2*,*3*). Reports have described reactivation of herpes virus infections after vaccination and after use of oral corticosteroids (*5*,*7*). HSV reactivation also has been reported following COVID-19 mRNA vaccines (*8*). In immunocompetent children, HZ has been linked to changes in cytokine profiles, similar to those seen after neurotropic viral infection, including infection with enteroviruses (*5*). At the time of HZ reactivation, this patient also was recovering from rhinovirus infection, which can suppress and dysregulate immune competence (*9*). We hypothesize that COVID-19 vaccination led to a shift in CD8 T-cell immunity, resulting in this unusual and rarely observed reactivation and dissemination. Immune dysregulation attributable to rhinovirus infection also might have exacerbated the immune shift after COVID-19 vaccination.

In conclusion, COVID-19 and varicella vaccines are extremely effective and safe in preventing disease in children. However, to ensure appropriate patient care, clinicians must be aware that rare sequelae, such as HZ reactivation, zoster meningitis, or both, might also occur.
